# Effect of Nitazoxanide, Artesunate Loaded Polymeric Nano Fiber and Their Combination on Experimental Cryptosporidiosis

**Published:** 2019

**Authors:** Enas Fakhry ABDELHAMED, Eman Magdy FAWZY, Said Mahmoud AHMED, Rabab Sayed ZALAT, Hayam Elsaid RASHED

**Affiliations:** 1. Department of Medical Parasitology, Faculty of Medicine, Zagazig University, Sharkia Governorate, Egypt; 2. Department of Zoology, Faculty of Science, Zagazig University, Sharkia Governorate, Egypt; 3. Department of Medical Parasitology, Theodor Bilharz Research Institute, Giza, Egypt; 4. Department of Pathology, Faculty of Medicine, Zagazig University, Sharkia Governorate, Egypt

**Keywords:** *Cryptosporidium*, Oxidative stress, Artesunate, Polymeric nanofiber

## Abstract

**Background:**

*Cryptosporidium parvum* is a dangerous intestinal pathogen due to its devastating effect on immunocompromised individuals. Considering low efficacy, high toxicity in addition to the development of resistance for the drugs used, this study aimed to find a new alternative treatment having the advantage of lower doses and minimal toxicity. We used a novel combination between artesunate loaded polymeric nanofiber (ALPN) and nanazoxide that had not been tried yet.

**Methods:**

Sixty Swiss Albino mice aged 6–7 wk, weighting 20–24 gm were used in Theodor Bilharz Research Institute (TBRI) Cairo, Egypt in 2017. *C. parvum* oocysts collected from patients were identified by polymerase chain reaction to be used for infecting animals. The effect of combination between ALPN and nana-zoxide were assessed by oocyst count in stool of experimental animals using modified Ziehl-Neelsen stain and histopathological changes in intestinal tissue. Antioxidant activity of nanofiber-loaded artesunate was estimated in serum, renal, hepatic and intestinal tissues by demonstrating the reactive oxygen species and the total antioxidant capacity. It was confirmed by detection of inducible nitric oxide synthase (iNOS) antibody.

**Results:**

The novel combination between ALPN and nanazoxidehas a harmonizing effect in reducing oocyst shedding (94.4%), the mean value of the antioxidant levels in liver, intestine, kidney, and serum were the highest level (10.15, 22.4, 6.22, 14.08 respectively) resulting in the decrease of oxidative stress in tissues. Marked improvement of histopathological features was obtained.

**Conclusion:**

This combination has a promising therapeutic effect against cryptosporidiosis particularly in immunocompromised individuals considering minor toxicity.

## Introduction

*Cryptosporidium* is an important protozoan human and animal pathogen (1) that is waterborne and foodborne infection (2). This pathogen causes self-limited diarrhea in immunocompetent patients and severe illness in immunocompromised (3). In Africa and Asia, *Cryptosporidium* infection is the second cause of severe diarrhea in young children (4). WHO report 2016 stated that cryptosporidiosis caused 18% of the deaths in children below 5 yr old (5).

Innate resistance to drug therapy by *C. parvum* has been demonstrated because of lack of specific targets at the molecular levels, difference in biochemical pathways, parasite’s unique location that affects drug concentration and finally the presence of pump efflux, which discard the drugs outside (6). Although nitazoxanide (NTZ) is the treatment for *Cryptosporidium* infections (7), it has limited efficacy in most cases. The need for safe and effective therapy is necessity (8). Therefore, we make a new trial by loading artesunate on nanofiber to investigate its therapeutic effects on experimental cryptosporidiosis. Plants of the genus *Artemisia* (family *Asteraceae*) are used as alternative antihelminthic in livestock (9,10). *A. annua* leaves are the viable source of artemisinin used to produce drugs such as artemether, arteether, and artesunate, which are effective against chloroquine-resistant malaria (11).

Anticancer drugs, enzymes and definite vitamins are bioactive molecules carried on nanofibers (12). They posses unique characteristics of higher loading efficiency for large protein drugs. To get effective treatment of different diseases, nanofibers were well used to transport the drug into the body organs (13). On the other hand, PEO (polyethylene oxide) has excellent biocompatibility, minimal toxicity and good solubility in water and other solvents. Therefore, it was used as a drug carrier (14).

The change in the balance between oxidant and antioxidant mediators in the cells is defined as the oxidative stress. It is attributed to excess production of reactive oxygen species (ROS). As natural defense, immune cells produce ROS that act as a double-edged sword. Although they are important for eradication of pathogens, overproduction of them can damages the immune cells themselves and host tissues as well. However, many infectious organisms establish pathogenicity by ROS production to counteract the host immune system. Consequently, ROS may contribute to host tissue damage in infectious disease using several mechanisms. This suggests the potential for utilization of ROS scavengers in controlling certain aspects of infectious disease and reduction of host tissue damage (15). The oxidant activity is confirmed in pigs infected with *C. parvum* (16). Antioxidants may benefit the host by decreasing both tissue damage caused by oxidative stress and thus will decrease the severity of symptoms and the developing of complications. Decreasing oxidative stress will also allow the body to sustain a viable immune system that is capable of eradicating the pathogen (17).

In our study, the effect of artesunate loaded polymeric nanofibers (ALPN) and its combination with nitazoxanide was evaluated for treatment of *Cryptosporidium*in in experimentally infected immunocopromized mice. The effect of target drugs was studied by oocyst count and histopathological changes in intestinal tissue. Moreover, antioxidant activity of nanofiber-loaded artesunate was estimated in serum, renal, hepatic and intestinal tissues confirmed by detection of inducible nitric oxide synthase (iNOS) antibody.

## Materials and Methods

This nonrandomized control-trial study was performed in Theodor Bilharz Research Institute (TBRI) Cairo, Egypt, in 2017.

### Mice

Swiss Albino mice aged 6–7 wk, weighting 20–24 gm were used. Mice were free from any parasitic infection as determined by examining their stools on three consecutive days by the formol-ether concentration technique and modified Ziehl–Neelsen stain.

### The Experiment

Sixty immunocompromised mice divided into six groups of 10 mice each: G I: infected control, G II: infected then treated with nana-zoxide, G III: infected then treated with artesunate, G IV: infected then treated with combination of nanazoxide and ALPN, G V: infected then treated with ALPN, G VI: uninfected control.

### Ethical Statement and Recruitment

Informed consent was taken from all patients before obtaining faecal samples. The study was approved by the Research Ethics Committee, Faculty of Medicine, Zagazig University. Dealing with the experimental animals were done according to the international valid guidelines and they were kept up under suitable circumstances at Schistosome Biological Supply Program (SBSP) animal house of TBRI.

### Oocyst

*C. parvum* oocysts were collected from patients attending to outpatient clinics of Zagazig University Hospitals and suffering from diarrhea or gastrointestinal troubles. Fresh fecal samples were collected from which direct smear, formol-ether concentration technique (18) and modified Ziehl-Neelsen stain (19) were done. Part of stool was stored at −20 °C to be identified by PCR to detect the presence of *C. parvum* in each sample. Isolation of DNA from stool was done by DNA extraction using (QIA amp DNA Stool Mini Kit QIAGEN, Hilden, Germany). Amplification of extracted DNA was done by using quantification of *Cryptosporidium* 18S ribosomal gene for general laboratory and research use only, according to manufacture instructions. The other part of stool oocysts were purified through discontinuous sucrose gradient flotation according to Suresh and Regh (20) for mice infection.

### Nanopolymer

Polyethylene oxide (PEO, Mv ∼ 900,000, Sigma–Aldrich) and artesunate drug (Ph. Int. from India by Ipca laboratories limited) were dissolved in Milli-Q water for 6 h (21). The used concentrations of PEO were 7%, 9% and 11% w/v and the drug concentration was 10% w/w respecting PEO final suitable concentration. The PEO and PEO/ artesunate solutions were then loaded individually (21). High voltage power supply (Spellman High Voltage Elec. Corp., MP Series) was used for the electrospunning of solutions at 0.3mm/min. The nanofiber scaffolds deposition, was done.

### Fabrication of PEO and PEO loaded with drug nanofibers

The process of nanofibers fabrication was performed by electrospin workstation (Ucalery, Beijing, China SS-25344, UC120815). The electrospun nanofibers were peeled off and kept in a dry place to remove the residual solvent.

### The characterization process Morphology

Field emission scanning electron microscopy (FE-SEM S-4800, Hitachi Ltd., Japan) at 10Kv was used for observing the morphology of the prepared nanofibers. Analysis of the obtained images was done by Image-Pro Plus 6.0 (Media Cybernetics, Inc., USA). The average and standard deviations were plotted. To establish a desirable set of conditions to produce electrospun PEO nanofibers. PEO were produced by electrospinning of the solution with high molecular weight. Poly ethylene oxide (PEO) dissolved in water by different concentration to evaluate the morphology and detect the suitable concentration of PEO used later (21). The effect of solution concentrations has investigated by the FE-SEM images. Different concentration of the PEO solutions (7%, 9%, 11%).

### Preparation of polymer solutions loaded with the drug

Electrospinning of PEO loaded with the drug was carried out at room temperature. The selected concentration of electrospun PEO nanofibers firstly and completely disappeared. Then 10 wt. % of the drug was added to the polymer solution and left overnight in a magnetic stirrer. Nanofibers collected from the aluminum foil then kept in vacuum for four hours.

### Drugs

Dexamethasone sodium phosphate in a dose of 20 μg/kg/d was used for immune suppression of mice. Prior to infection, it was injected intramuscularly three times/week for 20 successive days (22). Nanazoxid (Medizen pharmaceutical industries for Utopia pharmaceuticals) was given (100 mg/kg/d) for seven successive days. Artesunate (Ipca Laboratories, Sejvta, Ratlam, Kandivliind Estate) is given as 150 mg/kg/d for seven successive days. The dose of ALPN was 150 mg/kg for seven days per mice and the groups taken the combined drugs have been given half the dose of both nanazoxid and ALPN.

After 20 d of immune suppression, all groups were dead so, the experiment was repeated with the same dose but at lower duration of immune suppression (15 d only). Using oral gavage, all mice were inoculated with 10^3^ *Cryptosporidium* oocysts per mouse (23). The drug was given after seven days of infection and continued for seven days for scarification after three weeks.

### Parasitological examination

Twenty-one days after treatment, fecal samples were collected from each infected mouse. Stool samples were examined by modified Ziehl-Neelsen staining (19). The percentage reduction in oocyst counts (OC) was calculated as [mean OC of controls − mean OC of treated groups/mean OC of controls] × 100.

### Histopathological examination

Part of the duodenum was obtained from each mouse, fixed in 10% neutral formalin then embedded in paraffin. The obtained sections were stained with hematoxyline and Eosin.

### Reactive Oxygen Species and Total Antioxidant Capacity

Weighing and homogenization of jejunum, liver, and kidney were done for preparing 50% (w/v) homogenate containing 50 m M Tris-HCl and 300 m M sucrose. The supernatant was got from homogenate after centrifugation and 10% dilution in the Tris-sucrose buffer was obtained. Serum samples were collected and stored at −80 °C. The reagents used are the substrate (H2O2) (Dilute 1000 times before use) and chromogen, enzyme-cuffer.

### Procedure

The colorimetric technique using commercial kits (Biodiagnostic, Egypt) was used for measuring the total antioxidant capacity then was calculated as (A_B_-A_SA_ × 3.33) (24).

### Immunohistochemical examination of iNOS antibody

Immunostaining was performed on serial thin sections of paraffin blocks (4 μm thickness). Xylene was used to deparaffinize tissue sections. They were rehydrated in ethanol then treated with hydrogen peroxide 3% for 10 min to exclude nonspecific reaction. Microwave antigen retrieval was performed in citrate buffer 0.01 M (pH 6.0) for 15–20 min. The slides washed in phosphate buffer saline (PBS) were incubated with anti-iNOS antibody (dilution 1: 50; Santa Cruz Biotechnology, USA) for 60 min at room temperature. Binding site of primary antibodies was visualized by Dako En Vision ™ kit (Dako, Copenhagen, Denmark). The tissue sections were incubated in diaminobenzidine (DAB) for 15 min then counterstained with Mayer’s hematoxylin to visualize the immunohistochemical reaction. The evaluation of iNOS staining was done by assessment the proportion of cells expressing iNOS as a continuous percentage (0%–100%) scale using light microscopy at high magnification (× 40). A score of +3 was considered positive (25).

## Statistical analysis

Data were analyzed by SPSS software (ver. 19, Chicago, IL, USA). Test one-way ANOVA was used to calculate the significance between all groups but independent t-test used between every two groups. The results were considered statistically significant if the *P*-value was <0.05.

## Results

The lowest number of oocyst shedding (4.5±1.19) was detected in group IV. Consequently, the highest oocyst reduction percentage (94.4%) was obtained in the same group. A high statistically significant difference (*P*<0.001) present between group IV and group I, while a statistically significant difference (*P*<0.05) was obtained between groups II, III, V and group I.

The uninfected control Group VI had negative results in (Table 1). Concerning antioxidant capacity in liver, intestine, kidney and serum (Table 2), a highly statistically significant difference (*P*<0.001) was found between group IV and group I, whereas a statistically significant difference (*P*<0.05) was detected between groups II, III, V, and group I. There was also statistically significant difference (*P*<0.001) between all groups. Moreover, the mean value of the antioxidant levels in liver, intestine, kidney, and serum were at the highest level (10.15, 22.4, 6.22, 14.08 respectively) in group IV.

**Fig. 1:**
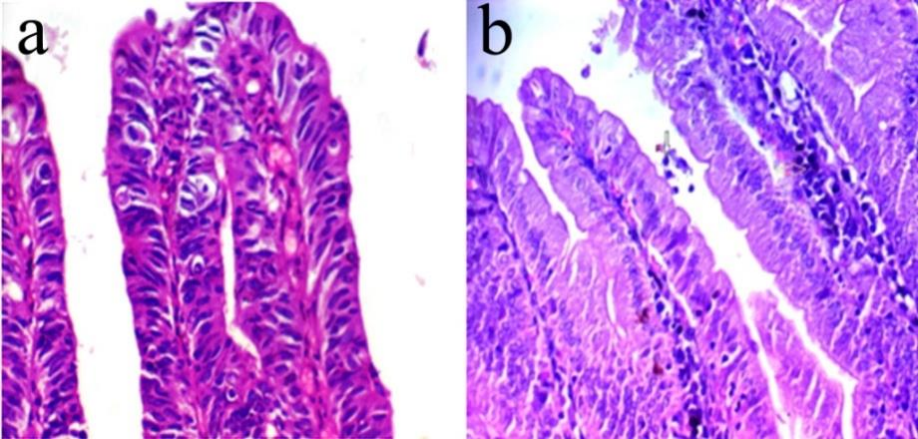
**(a–b)** Histopathological changes of intestinal epithelium in infected control immunocompromised group **a**-Intestinal epithelium showing high-grade dysplasia manifested by marked pleomorphism, absence of mucin, and frequent mitoses (arrows) (H&E ×400), **b**-showing presence of few parasites of *Cryptosporidium* at the brush border of epithelial cells of the villi (arrow) with infiltration of inflammatory cells (arrow) (H&E ×400)

The histopathological changes of intestinal epithelium in infected control group I revealed high-grade dysplasia manifested by marked pleomorphism, absence of mucin, and frequent mitoses compared to group IV, which demonstrated a few numbers of *Cryptosporidium* parasite at the brush border of the intestinal villi surrounded by inflammatory cells infiltration (Fig. 1a–b).

**Table 1:** The mean number and the percentage reduction of *Cryptosporidium* oocyst shedding in immunocompromised mice treated with artesunate loaded polymeric nanofiber

***Groups***	***(Mean±SD) (Range)***	***% Reduction in oocysts number***	***P value***
G I	81.75 ± 8.82 (70–90)		*P*1**
GII	73.3 ± 8.79 (39–80)	42.5	*P*2*
GIII	72.5 ± 8.75 (20–77)	57.2	*P*3*
GIV	4.5 ± 1.19 (3–6)	94.4	*P*4**
GV	75 ± 8.73 (55–80)	16.5	*P*5*

*P*<0.001** highly significant difference

*P*<0.05 * significant difference

*P*1 means statistical difference between 5 groups

*P*2 means statistical difference between G I versus G II

*P*3 means statistical difference between G I versus G III

*P*4 means statistical difference between G I versus G IV

*P*5 means statistical difference between G I versus G V

**Table 2:** The total antioxidant capacity in immunocompromised mice treated with artesunate loaded polymeric nanofiber

***Organs***	***GI***	***GII***	***GIII***	***GIV***	***GV***	***P value***
Mean ± SD (Range)						
Liver (Mm/g)	2.08±0.09 (1.8–2.9)	2.03 ± 0.08 (2.0–2.89)	3.5±1.85 (3.0–3.9)	10.15±1.15 (8.64–11.49)	3.33± 2.07 (2.65–8.64)	*P*1* P2 *P*3 * P4** P5 *
Intestine (Mm/g)	5.78 ± 0.26 (5.12–6.56)	6.12 ± 0.84 (5.42–7.57)	6.33 ± 0.69 (6.12–8.98)	22.4 ± 1.62 (20.6–24.7)	6.22 ± 0.53 (5.79–8.0)	*P*1** P2* P3* P4** P5*
Kidney (Mm/g)	1.27± 0.18 (0.89–1.9)	1.67± 0.38 (1.23–1.91)	2.34± 1.16 (2.13–4.98)	6.22± 1.65 (4.32–8.99)	1.94± 0.90 (0.99–3.23)	*P*1** P2** P3** P4 ** P5 *
Serum (Mm/l)	3.2 ± 0.29 (3.2–4.1)	3.78 ± 0.60 (3.23–4.54)	4.13 ± 0.67 (3.23–4.99)	14.08 ± 0.97 (12.7–15.3)	4.7± 0.23 (4.2–5.32)	*P*1** P2* P3* P4** P5*

*P*<0.001** highly significant difference

*P*<0.05 * significant difference

*P*1 means statistical difference between 5 groups

*P*2 means statistical difference between G I versus G II

*P*3 means statistical difference between G I versus G III

*P*4 means statistical difference between G I versus G IV

*P*5 means statistical difference between G I versus G V

**Fig. 2:**
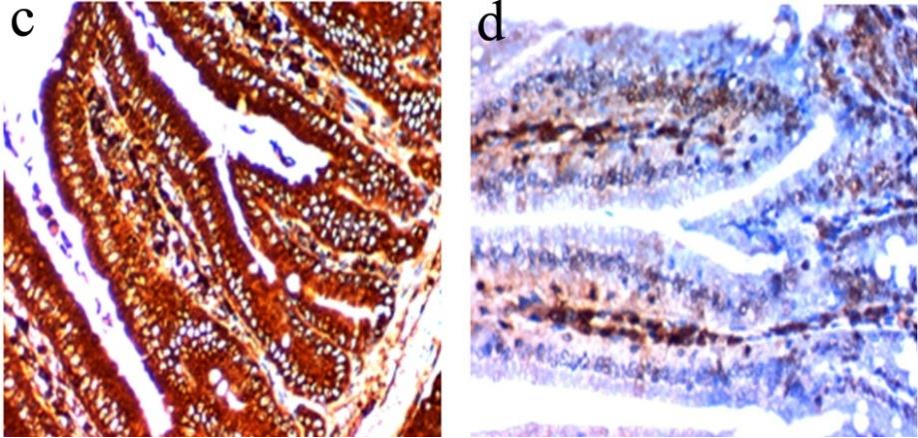
**(c–d)** Immunohistochemical changes of intestinal epithelium in infected control immunocompromised group compared to G: IVa (receiving combined treatment with nanazoxide and artesunate loaded nanofiber): **c**-Strong cytoplasmic expression of iNOS in the dysplastic intestinal epithelium infected by *C.parvum* (IHC ×400), **d**-Weak cytoplasmic expression of iNOS (IHC x400)

**Fig. 3:**
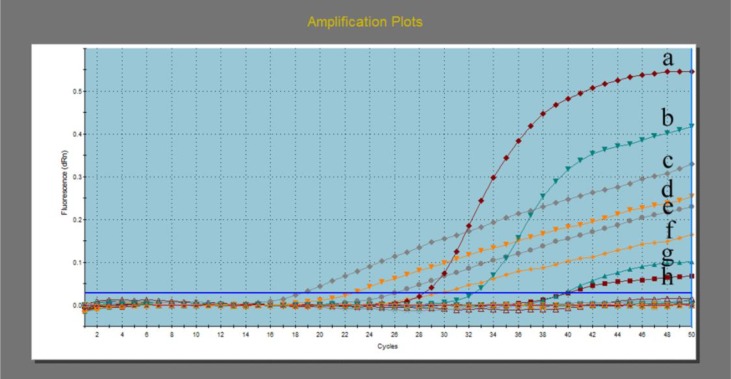
Amplification plot showing results of stool samples by real-time PCR assay. Plots (g, h) have fluorescent values above threshold line “horizontal blue line” so considered positive samples but with different Ct values. Plots (a, b) were ACTB controls while the remaining plots were amplified standards. N were negative samples

In this study, the drug artesunatum was used as a model antiparasitic drug in 11% PEO nanofibers and 10 wt. % of the drug. Meanwhile, the fibers prepared with 11% PEO was used as a control. FE-SEM observation showed that there were no obvious morphology and diameter differences between the nanofibers with or without drug. The average diameters of the 11% (w/v) drug-doped PEO were respectively 198±38, and 211 ± 40 nm for the 11% (w/v) and 10 wt.% drug-doped PEO nanofibers (Figs. 2, 3).

## Discussion

*Cryptosporidium* is considered a serious problem for infected individuals especially the immunosuppressed patients in developed and developing countries. In addition to the multiple adverse effects of the available drugs used for treatment, most of the immunodeficient patients did not respond to any of them (26). Consequently, new drugs against this parasite is urgently needed. In the present study, the therapeutic and the antioxidant effects of ALPN versus the traditional nanazoxanide treatment were evaluated. We compared the efficacy of artesunate, nanazoxide, ALPN and their combinations for treatment of experimental cryptosporidiosis in immunocompromised mice.

For drug delivery purposes using electrospun nanofibers, there is more demand to use biodegradable polymers. To the best of our knowledge, no studies have been reported on the efficacy of artesunate-loaded electrospun PEO nanofiber scaffolds. In this research, we investigated the synergistic effect of these polymers and the effect of adding artesunate drug in electrospinning and biological properties. The morphology of the nanofiber scaffolds was investigated. The electrospinning at relatively low polymer concentrations results in nanoparticles and fiber with beads rather than only fibers. Moreover, the nanoparticle formation process can be termed as electrospray. The polymer solution from the tip of a capillary begins to fragment into droplets at very low concentration. At concentration of 7% for PLA, the fibers disappeared and the complete spheres deposited on the surface of scaffolds. In high magnified image for 7% PEO concentration we can notice that the small fiber presents and the fiber diameter were 46±23 nm, and by increasing the polymer concentration from 7% to 9% or 11% the fiber diameters were increased significantly to 109±33 nm and 198±38 nm. About 9% and 11% PEO nanofiber morphology better than the other concentrations, so that the 11% PEO concentration used for loading the drug. However, if the polymer solution was more concentrated, the electrospinning process will be too difficult to eject the fluid jet from the needle (27).

The results of the present study indicated that the combination of both nanazoxide and ALPN had the best therapeutic effect on experimentally infected mice with *Cryptosporidium*. This combination presented in Table 1 showed the highest reduction rate (94.4%) of oocyst shedding in immunocompromised groups when compared with other infected groups. This result could be explained by the statement (28) that aqueous alcohol *Artemisia herba-alba* had a significant reduction of oocysts count of *Cryptosporidium* in experimentally infected mice. Moreover, a significant reduction of *C. andersoni* growth in vitro was reported at a concentration of 10 μg/ml NTZ that is similar to its inhibitory effect on the growth of *C. parvum* in vivo (29). However, a competent immune system is required to get rid of the parasite plus NTZ as a treatment (7). Similarly, a higher intensity for oocyst shedding was reported in immunosuppressed (30).

Measuring the total antioxidant capacity is more accurate than the sum of separate measurable antioxidants. It reveals the collective effect of all antioxidants present in liver, intestine, kidney, and plasma (31). In our research, the total antioxidant activity was estimated in liver, intestine, kidney, and serum of immunocompromised groups. The highest free radical scavenging capacity (antioxidant capacity) in liver, intestine, kidney and serum (Table 2) observed in group taken the combined treatment with nitazoxanide and ALPN (G IV) followed by group treated with artesunate and ALPN only (GIII). The least free radical scavenging capacity was observed in infected control (GI) followed by group treated with nitazoxanide alone (GII). There was highly statistically significant difference (*P*<0.001) between GIV and GI. Comparably, a significant increase was observed in lipid peroxidases (LPO) and reduction in levels of superoxide dismutases (SOD), catalases (CAT) and GSH in liver and intestine of mice group given dexamethasone (32). This oxidative stress induces free radical that plays an important role in the development of *C. parvum* infection in mice. In addition, *A. annua* water extract had great antioxidant activity (33).

Concerning histopathological examination of duodenum in immunocompromised groups, high-grade dysplasia was demonstrated in the intestinal epithelium. It was manifested by marked pleomorphism, absence of mucin, and mitosis in infected control group I (Fig. 1a). After therapy, histopathological examination showed few numbers of *Cryptosporidium* parasite at the brush border of epithelial cells of the villi with infiltration of inflammatory cells in group IV (Fig. 1b).

The immunohistochemical examination of iNOS antibody revealed strong cytoplasmic expression in the dysplastic intestinal epithelium of infected control group (Fig. 2c) confirming the strong oxidative stress done by the parasite when compared with weak cytoplasmic expression of iNOs group IV (Fig. 2d) that exhibit the effect of the drugs in lowering oxidative stress on tissue.

## Conclusion

Our study was unique as it was the first, which tried the combined treatment ALPN and its combination with nitazoxanide in immunocompromised mice. This combination had the best reduction rate of oocyst shedding and the highest total antioxidant activity. Consequently, it stimulates scavenger systems, which control the oxidative stress. This combination seems to be promising in reducing the devastating effect of *C. parvum* in immunocompromised individuals evidenced by marked improvement in histopathological features.
